# Physicians’ use of a digital tool with information views to support chart review of complex patient cases

**DOI:** 10.3389/fdgth.2026.1954602

**Published:** 2026-10-20

**Authors:** Amanda Emén, Torbjørn Torsvik, Gro Rosvold Berntsen, Marte Broks, Aslak Steinsbekk

**Affiliations:** 1 Department of Public Health and Nursing, Faculty of Medicine and Health Sciences, Norwegian University of Science and Technology (NTNU), Trondheim, Norway; 2 Norwegian Centre for E-health Research, Tromsø, Norway; 3 UiT the Arctic University of Norway, Institute of Community Medicine, Tromsø, Norway

**Keywords:** chart review, clinical workflow, complex care needs, digital support, EHR, navigation, person-centered, usability

## Abstract

**Background:**

Clinicians spend a substantial amount of time in electronic health records doing chart review. Chart review is particularly challenging in complex patient cases with several health challenges, involved professionals, organizations, and interventions, with a large volume of information stored in clinical notes.

**Objective:**

To investigate physicians’ use of a digital tool with information views that summarize and visualize patient information from several sources to support chart review of complex patient cases.

**Methods:**

This exploratory descriptive study used data from the intervention arm of a randomized controlled trial evaluating a digital tool called DigiTeam. The tool contained 10 information views: Overview, About the Patient, Medical History, Clinical Notes List, Involved Health Professionals, Medication List, Municipality, Diagnoses GP, Diagnoses Hospital, and Health Problem Summaries. Twenty-nine physicians used DigiTeam to review three complex patient cases. All interactions with the tool were logged. Descriptive statistics were used to analyze chart review time, use of information views, navigation patterns, switching between information views, and use of clinical notes and hyperlinks.

**Results:**

Mean chart review time was 24 min and 20 s. The largest proportion of chart review time was spent in About the Patient (24%), which presents information on what matters to the patient and their social and contextual situation, followed by the Clinical Notes List (20%) and Health Problem Summaries (18%). After starting on the Overview page, About the Patient was opened in 71% of the chart reviews, followed by Medical History. Usage patterns were similar across patient cases. On average, physicians opened 8.3 clinical notes and 2.9 hyperlinks per chart review. Switching between information views remained relatively stable throughout the review process (mean 0.75 switches per 5% of chart review time).

**Conclusion:**

When reviewing complex patient cases, physicians mainly combined information views that summarize and visualize patient information with reading clinical notes. The most used information views included information on the patient's context, social situation, what matters to the patient, the medical history, and specific health problems. These findings, based on actual use rather than perceived usefulness, suggest that integrating such elements within digital tools may better support chart review for patients with complex and long-term care needs.

## 1 Introduction

Clinical chart review is a core task performed by clinicians (1, 2). It involves searching, selecting, and synthesizing patient information to form a basis for further decision-making. This work is currently mostly performed in electronic health record systems (EHRs). Clinicians spend a substantial portion of their time in EHRs (3–5), of which approximately 30%–40% is devoted to chart review (1–4, 6). As the volume of patient information increases, it becomes increasingly difficult to gain an overview (7). Consequently, chart review is particularly challenging in patients with complex, long-term care needs, with multiple health problems, who receive care from professionals at different health services.

Although functionalities in the EHRs support many tasks within a clinician's workflow, including chart review, many studies report challenges of information overload, limited access to relevant information, scattered information, and excessive clicking and scrolling (8–10). These challenges influence how clinicians experience and navigate the EHR to build an overview (11). Studies show that clinicians frequently switch between different parts of the system (12, 13), visiting on average 6.3 screens per patient (13). Such navigation reflects the need for clinicians to piece together fragmented information (12, 14) and check information across sections to confirm their understanding of the patient's situation (12, 15).

Clinicians’ navigation is guided by clinical reasoning processes and information needs. This involves identifying active health problems, determining diagnoses and causes, and establishing a plan (16). Key information is chief complaints, urgent vs less urgent problems, interactions between current issues, the patient's medical history, and already ongoing plans initiated elsewhere (13, 17–20). Rather than proceeding linearly through the record, clinicians tend to prioritize sections that offer a concise overview to orient themselves to the patient's situation (18, 21).

This is reflected in how clinical notes are read (18, 21–23). Studies show that clinicians spend a substantial proportion of their time in a limited set of sections, particularly clinical notes (2, 4, 19, 24), while also relying on other sections, for example, those that provide laboratory, imaging, diagnostics, and the overview page of the patient's chart (13). Furthermore, some sections are preferred to be used in combination, such as the medication list and problem list (25). As clinicians navigate between sections, they relate current status to prior information, balancing the need for a quick overview with the patient's history (26). Despite these common screen uses, clinicians’ navigation behavior varies, as shown in studies that found that the sequences of screens accessed during chart review vary (13, 27), reflecting differences in clinical roles, tasks, and reasoning strategies.

To support clinicians’ clinical reasoning and decision-making, the information should be summarized and presented for the clinical task. A recent systematic review found that snapshot-based summaries can facilitate rapid assessment of a patient's current status and prioritization of active issues, and timeline-based visualizations are suitable for understanding disease trajectories and longitudinal trends (28). Similarly, other studies have developed digital tools to address usability issues in EHRs and ways of presenting information (7, 25, 29–35). These studies presented solutions using text summaries and visualizations such as problem-oriented overviews, timelines, and representations of the frequency of health problems over time. It varies somewhat how these solutions have been tested, but broadly speaking, the focus has been on arguing for design concepts and using usability outcomes.

However, even if clinicians find a single design or information element or a package of elements useful and effective, they might not use them in practice. This highlights the importance of exploring actual use. One approach is to collect log data on how the tool or solution is used, which can reveal detailed usage patterns and show how clinicians navigate and interact with digital tools (36). Previous research, including both reporting from a systematic review (37) and individual studies (1, 2, 6, 15, 27, 38), has applied log data to examine various aspects of EHR usage.

However, no study has been identified that specifically investigated how physicians use and move between information views in digital tools when reviewing complex patient cases.

Therefore, this study aimed to describe physicians’ use of a digital tool (DigiTeam) with information views that present summarized and visualized patient information during the chart review of three complex patient cases. The following research questions (RQs) were addressed:

RQ1: How much time did physicians spend on the chart review overall, and how was this time distributed across different information views in DigiTeam?

RQ2: How did physicians use and navigate information views in DigiTeam at the start of the chart review and across the entire chart review process?

RQ3: How frequently did physicians switch between information views during the chart review, and did switching frequency vary across different stages of the review?

RQ4: How many original clinical notes and hyperlinks did physicians open during the chart review?

## 2 Materials and methods

### 2.1 Study design

This was an explorative descriptive study using data from the intervention arm of a randomized controlled trial (RCT) conducted between June and December 2024, of which more details of the methods and parts of the secondary outcomes are described in a manuscript submitted after a minor revision (Same authors, Title “The effect on usability of including information elements in a digital tool to support physicians’ clinical workflow in complex patient cases. A randomized controlled trial.”).

### 2.2 Ethics and privacy

This study was conducted in accordance with the Declaration of Helsinki (2013). Ethical approval was waived by the Regional Committee for Medical and Health Research Ethics (application no. 251632). The project was approved by the data protection officer at the University Hospital of North Norway (# 2760). The participants, and the patients sharing their medical records, received verbal and written information about the study and gave written voluntary consent.

### 2.3 Trial setting

This study was conducted as part of a larger project that developed a digital tool, called DigiTeam, designed to support chart review based on key quality-of-care principles for person-centred, integrated, and proactive care for patients with complex and long-term care needs (39) and investigated its effect in a three-armed RCT (submitted for publication). In the RCT, the participants were asked to use the allocated program version to write a summary note of the patients’ situation and care plans for each of the three patient cases. All participants completed the three chart reviews during a single session in a test environment outside clinical practice hours. This was intended to provide an uninterrupted setting in which participants could focus on the tasks and take the time needed to complete each chart review without distractions. The participants could participate either remotely or in-person, and DigiTeam was accessed through a secure cloud service provided by the University of Oslo. There was no time limit, but the session to complete the three patient cases was allocated 2 h.

### 2.4 Participants

The participants in this study were those allocated to the intervention arm in the RCT. The eligibility criteria for the RCT were to be a medical doctor who had graduated within the last 5 years or had experience working as a general practitioner (GP). Participants were recruited through the researchers’ network, social media, and the research group's website.

### 2.5 The digital tool “digiTeam”

DigiTeam is organised into 10 information views (pages): Overview, About the Patient, Medical History, Clinical Notes List, Involved Health Professionals, Medication List, Municipality, Diagnoses GP, Diagnoses Hospital, and Health Problem Summaries. Seven information views present information in ways not commonly found in traditional EHRs, while three information views (Clinical Notes List, Diagnoses GP, and Diagnoses Hospital) are typical views in EHR systems.

Information elements are used to build the information views. Information elements are defined as presenting information either by 1) textual summaries to summarise patient information or 2) visualizations to visualise development over time, both presented to support understanding of the longitudinal care process.

Interactive features are used in the information elements to support navigation, including access to more details. The interactive features in DigiTeam are hyperlinks for opening original clinical notes, clickable boxes or symbols, and timeline sliders.

When opening DigiTeam, the information view called the Overview page is the first to appear (Figure 1). This includes three information elements: 1) *The patient's voice* (Figure 1.A1), which summarizes what matters to the patient, short and long-term goals, and what the patient wants health professionals to know, 2) a short summarized overview of the patient's medical history across conditions (Figure 1.A2), and 3) a list of health problems on a timeline displaying when key healthcare contacts had occurred concerning the different health problems (Figure 1.A3).

**Figure 1:**
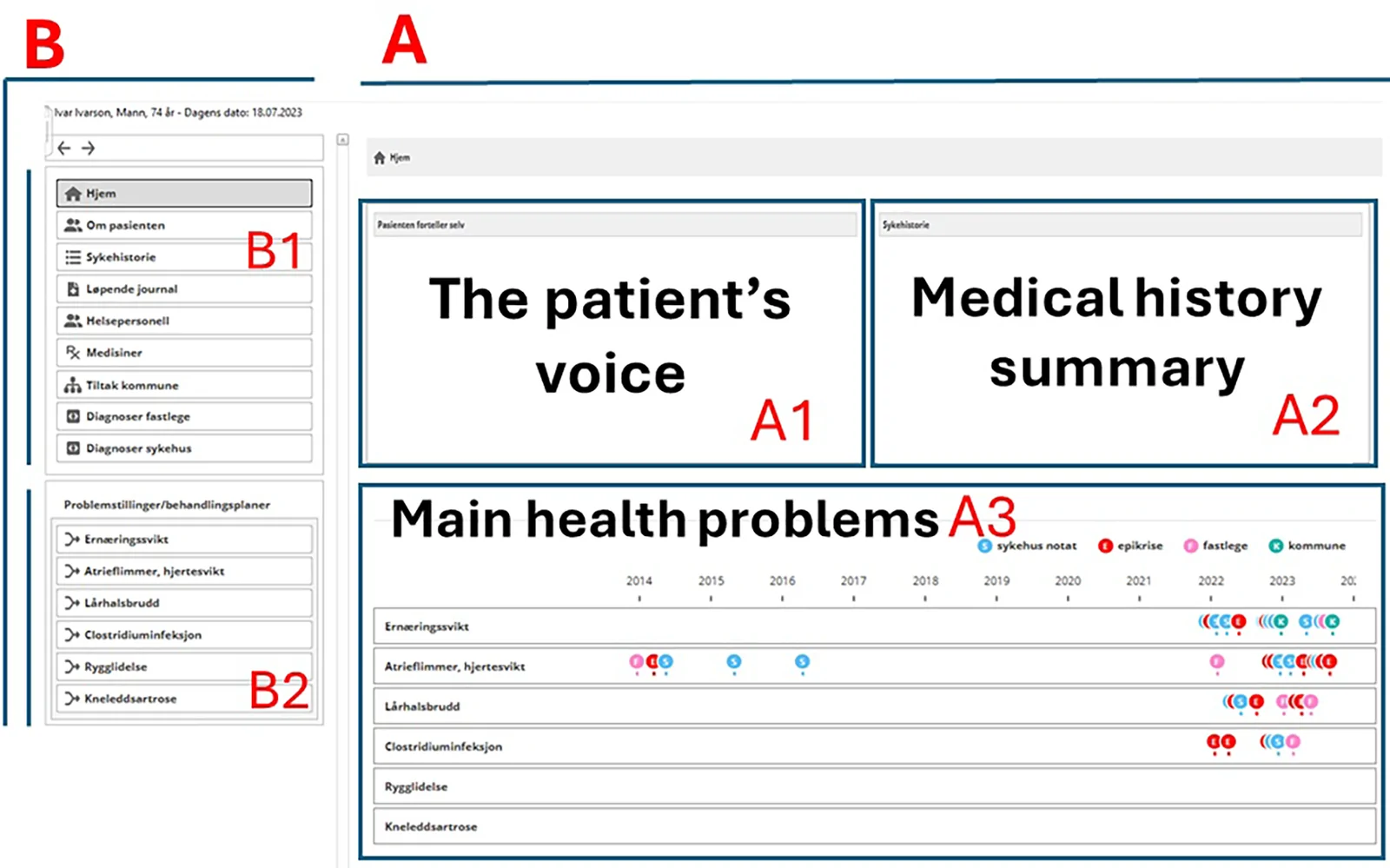
The overview page in DigiTeam. The sidebar menu (B) links to information views in DigiTeam (B1) and summaries of health problems (B2). The B1 section starts with the Overview page, which is displayed in section (A), containing the patient voice (A1), brief medical history (A2), and main health problems (A3). Adapted from “The start page of DigiTeam” by Amanda Emén, Marte Broks, Torbjørn Torsvik, Gro Rosvold Berntsen and Aslak Steinsbekk, licensed under CC BY 4.0.

The other information views are accessed via the sidebar menu (Figure 1, section B). These are: About the Patient, which provides more information about what matters for the patient, and information about family, social circumstances, identified needs, and a list of municipal health and social care services. The Medical History presents key medical events from all conditions, organized by time with the most recent event first. Clinical Notes List provides a chronological list of all available notes, with filtering and search functions. Involved Health Professionals display the roles and organizational affiliations of health professionals involved in the patient's care, grouped by health problem. The Medication list is grouped by current, previous, and as-needed medications. The Municipality offers an overview of municipal health and social care services. Diagnoses GP are diagnoses recorded in general practice (ICPC codes), and Diagnoses Hospital are diagnoses from specialist care (ICD-10 codes), both organized in order of time with the latest first. Health Problem Summaries (accessed from A3 and separate choices in B2) are summaries of specific health problems, with current state, care history, and plans.

Information about what matters to the patient and about the patient was collected from clinical notes and through patient interviews conducted as part of the DigiTeam development project and was included with the patients’ consent. The information displayed in DigiTeam was summarized primarily by a physician (author MB). The summaries were prepared before the RCT as part of the intervention content available to participants during chart review. The summarized information was not validated by the patients.

Figure 2 shows an example of an information view with a summary of a health problem, which includes hyperlinks to clinical notes that are the source of the clinical information in the summary (e.g., a GP note). The information views with elements that summarize information have a similar layout and function.

**Figure 2:**
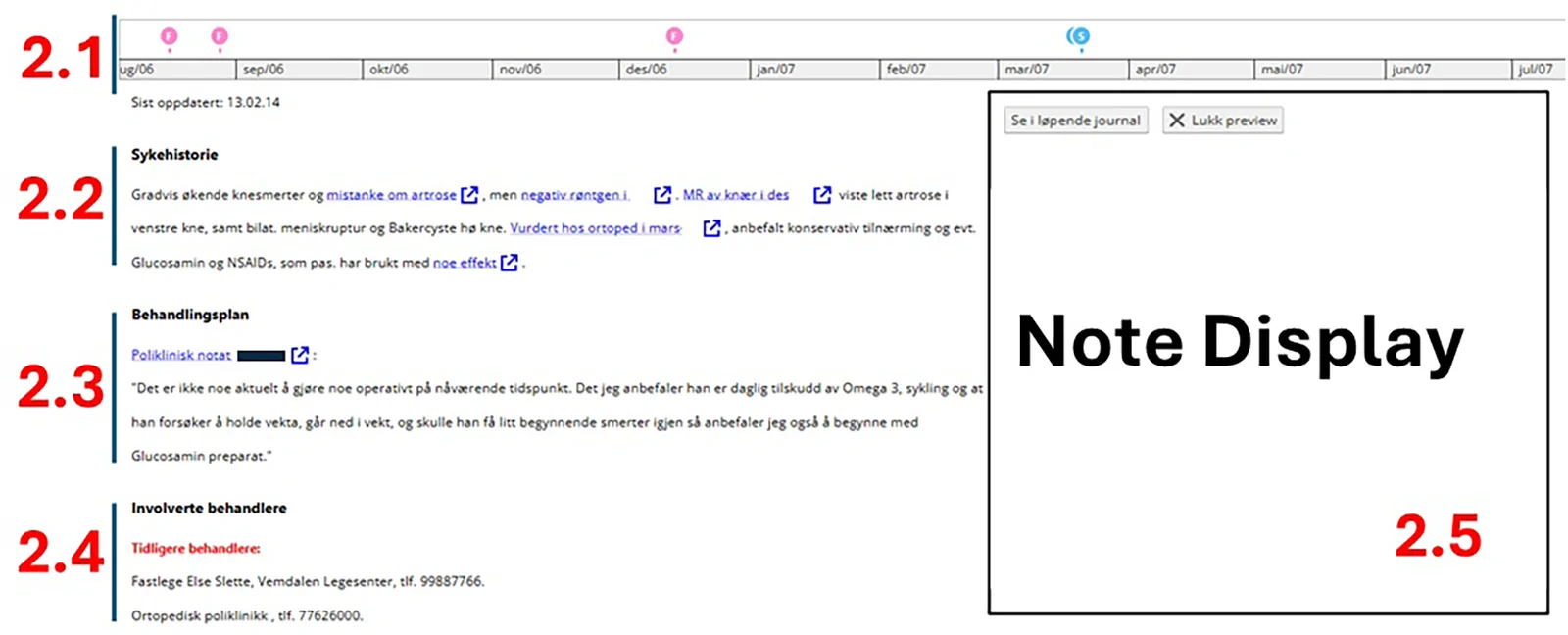
One example of an information view summarizing information about a health problem. This includes a timeline for the main healthcare contacts on the top (2.1), followed by the summary of the history of the complaint (2 2), treatment plan (2.3), and involved health professionals (2.4). The blue hyperlinks in the summary provide access to the clinical note from which the information was taken, and the note is displayed on the right side (2.5) when a link is clicked on. Adapted from “Example of a summary page” by Amanda Emén, Marte Broks, Torbjørn Torsvik, Gro Rosvold Berntsen and Aslak Steinsbekk, licensed under CC BY 4.0.

DigiTeam includes clinical notes from up to three different sources: general practice, specialist care, and municipal health and social care services, depending on the patient's case. The notes were categorized for analysis purposes into the types described in Table 1.

**Table 1:** Description of the clinical notes categories.

Source	Note type	Description
Specialist care	Discharge summaries/outpatient summaries	These documents include formal reports written after hospital admissions or outpatient encounters. They typically contain diagnoses, treatments, key clinical findings, and follow-up plans.
	Progress notes	Clinical notes documenting patient assessments, follow-up, treatment, and changes in health status during ongoing care.
	Letters to Patient	Communication addressed to patients, such as admission letters, appointment notifications, rescheduled or follow-up appointments, and waiting list updates.
	Admission notes	Initial clinical assessments written when a patient is admitted to the hospital, documenting presenting issues, early findings, and initial management plans.
	Nursing notes	Documentation created by nursing staff, including day, evening, and night reports; outpatient nursing notes; specialist nurse reports; and nursing admission or transfer notes. These notes describe observations, care activities, and clinical changes.
	Lab reports	Recorded laboratory results and related diagnostic information.
	Other clinical documents	Less frequent documents including diagnostic findings from imaging, medication-related information, administrative communications, and exchanges of clinical information between healthcare providers.
Municipal health and social care	Municipal notes	Documentation generated by municipal healthcare services, such as home care and nursing services.
GP	Out-of-hours primary care service	Documentation describes assessments, interventions, and clinical decisions made during urgent patient encounters outside regular GP hours.
	General practitioner notes	Notes written by the patient’s GP, representing ongoing primary care follow-up and medical management.

### 2.6 Task: chart review

All participating physicians were tasked with conducting a chart review of the same three patient cases, received in the same order. The physicians were asked to write a summary of the patient's situation, including medical status, social and personal factors contributing to the situation, what matters to the patient, and a follow-up plan including who should be involved in the plan.

The three patient cases were selected for their variation in medical complexity and care histories. They contained, respectively, 997, 1863, and 3050 PDF pages of clinical notes spanning up to 27 years from general practice, specialist care, and, for cases 1 and 2, also from municipal health and social care services. The main health challenges are:

- Case 1: A 74-year-old man with advanced multimorbidity with multiple acute hospital admissions, chronic hypotension secondary to heart-failure treatment, and nutritional decline. Receives daily municipal services.
- Case 2: A 44-year-old man with complete quadriplegia resulting from a cervical spinal cord injury sustained in adolescence, with multisystem involvement. Lives in municipally supported housing with 24-hour personal assistance.
- Case 3: An 82-year-old man with multiple chronic conditions, with no municipal services, who is the primary caregiver for his wife with Alzheimer's disease.

### 2.7 Data collection

Data was collected in logfiles for each of the chart reviews, using Apache log4j (40). The logfiles included timestamps for each interaction in the tool (Figure 3). To extract actions and timestamps from the logfiles, a custom Python script was developed using PyCharm (version 2024.2.4; JetBrains, Amsterdam, the Netherlands).

**Figure 3:**
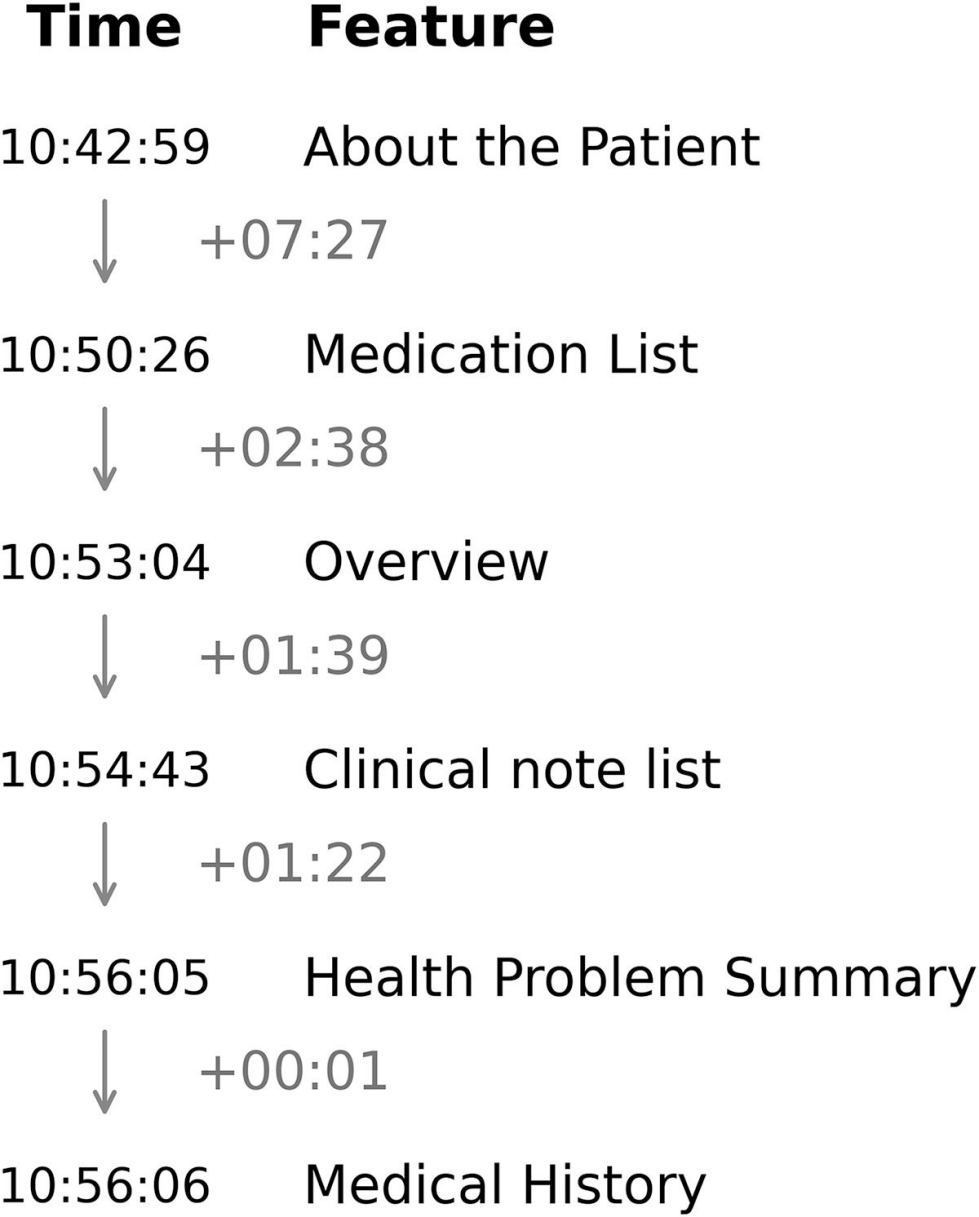
Simplified illustration of a logfile. Each timestamp marks when the participant interacts with the tool. The arrows, which were not part of the log files, indicate movement from one information view to the next, and the accompanying timestamps are the calculated time within an information view. In this example, the participant first spent 7 min and 27 s in About the Patient before spending 2 min and 38 s in the medication list.

### 2.8 Variables

For variables reporting “time spent,” the measure represents the logged interval during which the view remained open and does not capture what the participant was doing during that interval, such as reading, writing, or thinking.

To address RQ1, time spent during the chart review was collected for the whole review and for each individual information view:

**RQ1.A Total chart review time:** Time required to complete the chart review using DigiTeam. It was calculated as the elapsed time (minutes:seconds) from the first timestamp recorded when the overview page was opened at the start of the chart review until one timestamp before the last recorded action before termination of the program.

**RQ1.B Time spent on each individual information view:** Calculated as the interval from when an information view was opened until the next information view was opened. This was included to explore whether clinicians engaged more with certain information views when reviewing complex patient cases. For the health problems, these had their own information view but were counted as one information view in this study. For each chart review, the total time spent in each information view and its proportion of the total chart review time were calculated.

To address RQ2, usage patterns were operationalized through three measures:

**RQ2.A Usage patterns at the start of the chart review:** Describes which information views in DigiTeam were opened in the first five steps of the chart review. This was to explore what information was looked at initially. All participants started on the overview page (Step 0). Steps 1 to 4 were the next four information views that were opened. A filtering of ≥ 5 s was chosen as the minimum time the information view had to be open in steps 1–4 to be counted as viewed. The time spent and the proportion of chart reviews in each information view for each step were calculated.

**RQ2.B Overall usage patterns – transitions between information views:** This measure describes how users navigated from one information view to another within each chart review. This was to explore common sequences of information views opened and patterns in how these were combined. One transition was counted as going from one information view to another (e.g., Overview → About the Patient). For each transition pair, both the total number of transitions and their proportion of the overall number of transitions were calculated.

**RQ2.C Overall usage patterns – when each information view was used:** Describes which information views participants had opened at different points in time. At each time point, the proportion of chart reviews in which each information view was open was calculated; chart reviews in which DigiTeam had been terminated were classified as *No Record*. Open information views were visualized using both absolute time (minutes from the start to finish of the chart review) and relative time (percentage of each chart review's total duration to account for variation in overall chart review length).

To address RQ3, information view switching behavior was examined with one measure:

**RQ3 Frequency of information view switching over time:** Describes how often switching from one information view to another during the chart review occurred. This was to explore whether information view switching occurred more or less frequently at different stages of the chart review, such as at the beginning, middle, or end. Switching behavior was quantified by dividing each chart review's total duration into consecutive segments of 5% of the review time and counting the number of information view switches occurring within each segment. The proportion of chart reviews with at least one switch within each segment was also calculated.

To address RQ4, the opening of original clinical notes and the use of hyperlinks were examined at the two levels:

**RQ4.A Opening of original clinical notes:** Describes how many original clinical notes were opened, and which types of notes were accessed during the chart review. Original clinical notes were classified by type (see Table 1), and a note was counted as opened if it was displayed for more than five seconds. This was used to explore how many clinical notes were opened during each chart review, as the participants could choose to rely only on the summaries.

**RQ4.B Use of hyperlinks:** This measure describes how many hyperlinks were opened to access additional information during the chart review. Hyperlink use was quantified as the total number of hyperlinks clicked on in each chart review.

### 2.9 Data analysis

Descriptive statistics were used to answer all RQs. The analyses were performed in StataNow/MP version 19.5 (StataCorp LLC, College Station, TX, USA) for descriptive statistics, and PyCharm (version 2024.2.4; JetBrains, Amsterdam, the Netherlands) using the Python packages *pandas*, *seaborn*, and *matplotlib* for visualizations in addition to descriptive analyses.

The three chart reviews completed by each participant were included as separate observations in the main analysis. In addition, separate analyses were conducted for each patient case for the outcomes in RQ1 and RQ4.

Tables were used to answer RQ1 and RQ4, heatmaps and graphs were used to answer RQ2, and histograms were used to answer RQ3 and RQ4. Means and standard deviations (SD) are reported for relevant variables for all RQs. Results are reported using both absolute counts and proportions. Absolute counts of chart reviews and transitions are reported for RQ2. Proportions of chart reviews are reported for RQ2 and RQ3, and proportions of time are reported for RQ1–RQ3.

## 3 Results

Among the 29 physicians participating, 19 were female (66%), their mean age was 34, they had a mean of 7.1 years of clinical practice, and 8 were GP specialists (28%) (Table 2).

**Table 2:** Participant characteristics (*N* = 29).

Characteristics	Physicians N(%) or mean (SD), range
Female, *n* (%)	19 (66%)
Age years, mean (SD), (range)	34 (9.6) (26 to 60)
Years in clinical practice, mean (SD), (range)	7.1 (9.1) (0 to 33)
*5 years or more, n (%)*	10 (35%)
General practice specialist, *n* (%)	8 (28%)
Self-reported **better** EHR Skills than others, *n* (%):	9 (31%)
Weekly clinical experience with at least one of the patient cases, *n* (%):	20 (69.0%)
*Patient case 1*	17 (58.6%)
*Patient case 2*	4 (13.8%)
*Patient case 3*	14 (50.0%)

### 3.1 RQ1: time spent on chart review overall, and time on information views

The mean time spent on the chart reviews was 24:20 min, ranging from 9:26 to 56:38 (Table 3). Most of the time was spent on About the patient (24%; 5:30), followed by the Clinical Notes List (20%; 5:03), and the Health Problem Summaries (18%; 4:32) (Table 3).

**Table 3:** Mean (SD) total time spent in all cases combined and for each patient case, sorted by the mean proportion (mean %) for all cases. .

Information view	Mean % (SD) Mean time, mm:ss (SD)			
	All cases combined (*n* = 87)	Case 1 (*n* = 29)	Case 2 (*n* = 29)	Case 3 (*n* = 29)
About the Patient	23.7 (13.34) 05:30 (03:40)	24.9 (11.02) 08:01 (04:13)	25.3 (16.44) 04:38 (02:53)	21.0 (11.97) 03:54 (02:20)
Clinical Notes List	20.3 (18.53) 05:03 (05:28)	23.1 (17.15) 07:58 (06:42)	16.6 (16.16) 03:24 (03:31)	21.0 (21.84) 03:50 (04:41)
Health Problem Summaries	17.6 (18.37) 04:32 (05:09)	15.1 (14.79) 05:11 (04:58)	19.2 (19.82) 04:11 (05:01)	18.6 (20.37) 04:16 (05:34)
Overview	17.2 (15.23) 04:15 (04:06)	14.9 (12.45) 05:15 (04:58)	20.3 (18.06) 04:02 (03:35)	16.4 (14.71) 03:29 (03:33)
Medical History	12.1 (12.29) 02:39 (02:34)	11.3 (8.72) 03:37 (02:56)	12.8 (13.69) 02:11 (02:06)	12.3 (14.12) 02:09 (02:25)
Medication list	5.0 (8.14) 01:14 (01:50)	5.3 (6.67) 01:55 (02:22)	2.5 (3.83) 00:30 (00:42)	7.3 (11.51) 01:18 (01:49)
Municipality	1.2 (2.50) 00:22 (00:56)	2.1 (3.81) 00:45 (01:29)	1.2 (1.38) 00:17 (00:27)	0.5 (1.25) 00:05 (00:12)
Diagnosis Hospital	1.0 (2.47) 00:14 (00:37)	0.9 (2.02) 00:17 (00:36)	0.6 (1.51) 00:07 (00:15)	1.6 (3.45) 00:21 (00:51)
Diagnosis GP	0.9 (3.56) 00:14 (00:54)	1.6 (5.88) 00:28 (01:30)	0.5 (1.10) 00:09 (00:22)	0.6 (1.50) 00:08 (00:18)
Involved Health Professionals	0.8 (2.74) 00:12 (00:44)	0.7 (1.62) 00:11 (00:30)	1.1 (2.93) 00:19 (01:02)	0.8 (3.43) 00:08 (00:36)
All information views %	100%	100%	100%	100%
*Mean Time (SD), range*	*24:20 (11:04),* *09:26 to 56:38*	*33:36 (10:53),* *10:52 to 56:38*	*19:46 (07:23),* *09:26 to 38:41*	*19:38 (08:21),* *09:49 to 44:48*

There were no noteworthy differences overall in the proportion of time spent in each of the information views between the cases (Table 3). Case 3 did not have any municipal services, which explains the low use of that information view.

### 3.2 RQ2: use of information views at the start and during the entire chart review

#### 3.2.1 Initial use

The mean time spent in the first five steps of each chart review was 10:43 min (SD 4:56, Range 3:13 to 23:57), which is 44% of the total chart review time. The mean time spent in each view was similar, ranging from 01:59 to 02:19 min (Figure 4). They spent 2 min and 11 s on the Overview page (Step 0), which included What matters to the patient, a summarized medical history, and a health problem list with a timeline.

**Figure 4:**
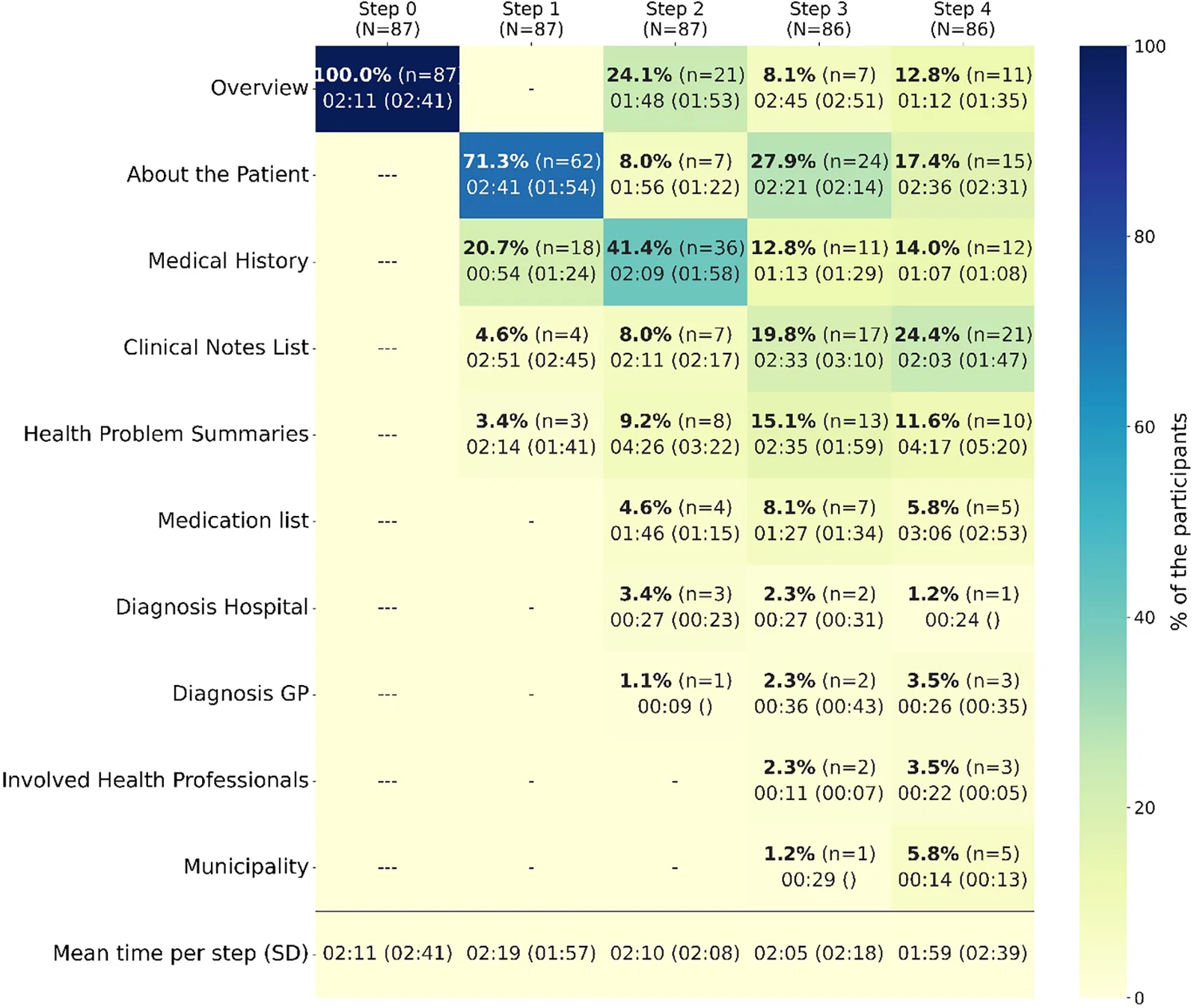
Heatmap over the first five steps when navigating in digiTeam for all cases combined. Proportion (number) of chart reviews and mean (standard deviation) time per information view in minutes: seconds.

In Step 1, after starting on the Overview page, About the Patient was opened in 71% of the chart reviews. In Step 2, Medical History was opened in 41% of the chart reviews, while the Overview page was returned to in 24% of the reviews. From Step 3 onward, navigation patterns became more varied. However, the use of Health Problem Summaries and the Clinical Notes List increased compared to the earlier steps.

#### 3.2.2 Overall usage patterns

Transitions between 90 different pairs of information views were possible. In total, there were 1311 transitions between information views across all chart reviews. The mean number of transitions per chart review was 15.1 (SD 8.4; range 2 to 39). The two most common transitions were from Overview to About the Patient (8.8%, 115 transitions) and from About the Patient to Medical History (8.0%, 105 transitions) (Figure 5).

**Figure 5:**
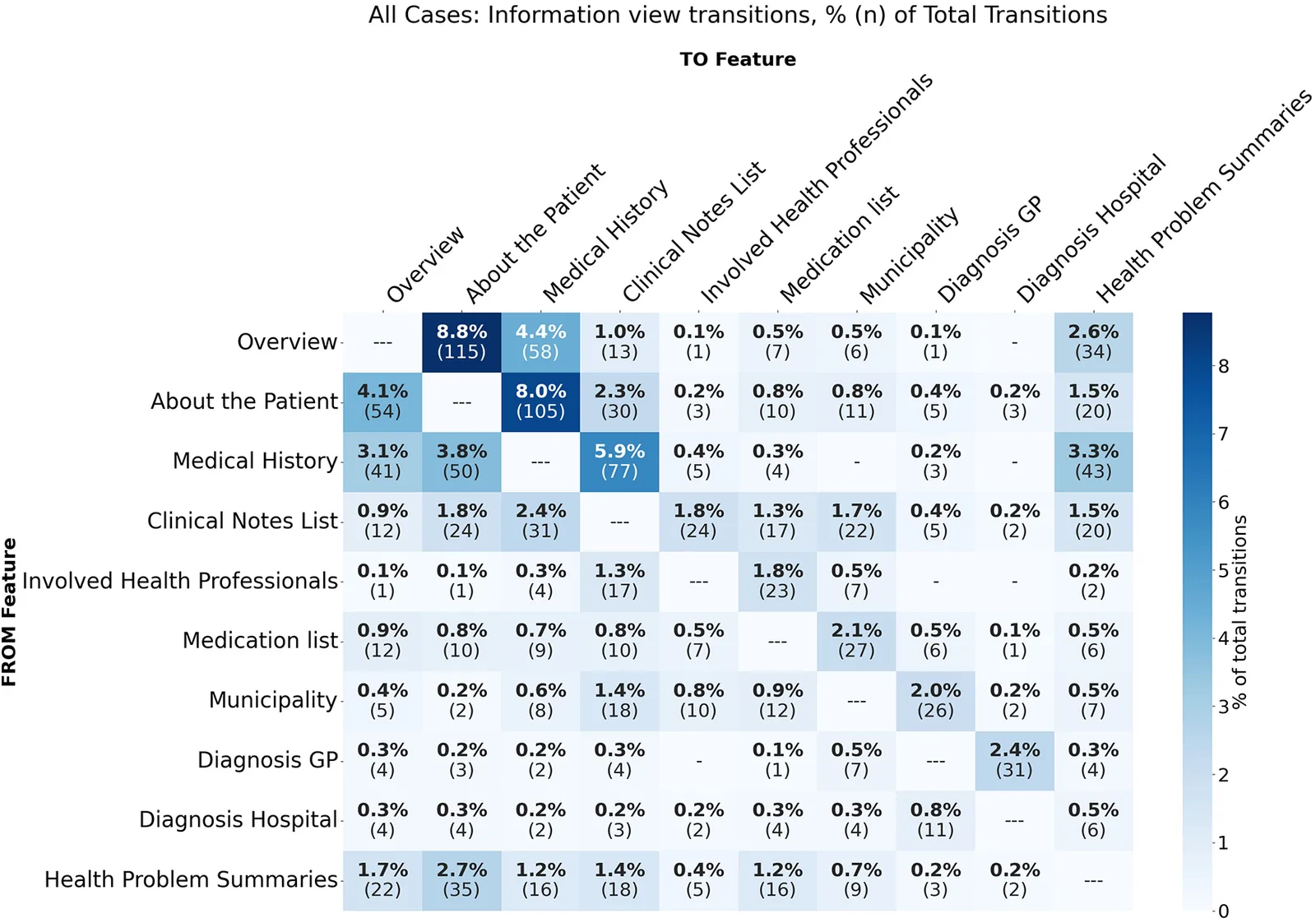
Information view transition heatmap based on all 1311 transitions between information views. Percentage of all transitions from the information view on the *y*-axis to the information view on the *x*-axis sorted in the order of the sidebar menu choices.

To illustrate changes in the use of information views, Figure 6 shows the proportion of chart reviews in which each information view was used at any point during the time spent reviewing the chart (Figure 6, A) and the relative time (proportion of total time spent) (Figure 6, B).

**Figure 6:**
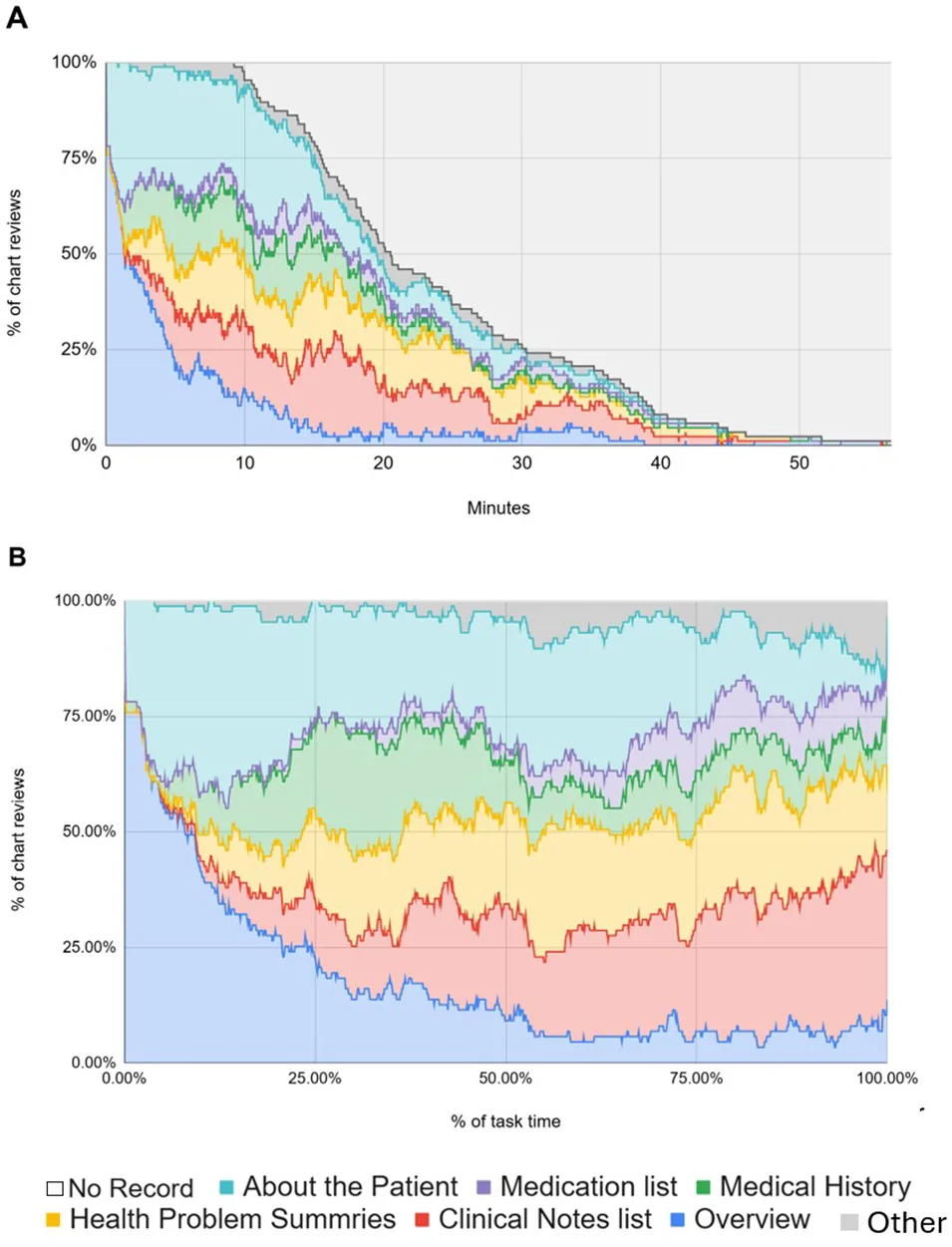
Percentage of chart reviews in which the different information views were used during chart review. Graph **(A)** (above) shows information view usage plotted against the absolute mean chart review time (0–57 min). Graph **(B)** (below) presents information view usage on a normalized time scale (0–100%). The category “Other” combines pages with little use for clarity (Municipality, Involved Health Professionals, Diagnosis GP and Diagnosis Hospital).

### 3.3 RQ3: switching between information views

The histogram (Figure 7) shows that the number of switches from one information view to another was stable, with a mean of 0.75 (SD 0.42) switches every 5% of the chart review time. There was a slight increase in the last 5% segment, with 1.5 (SD 2.3) switches. The average proportion of chart reviews with at least one switch in each 5% segment was 37.6% (SD 13.6%).

**Figure 7:**
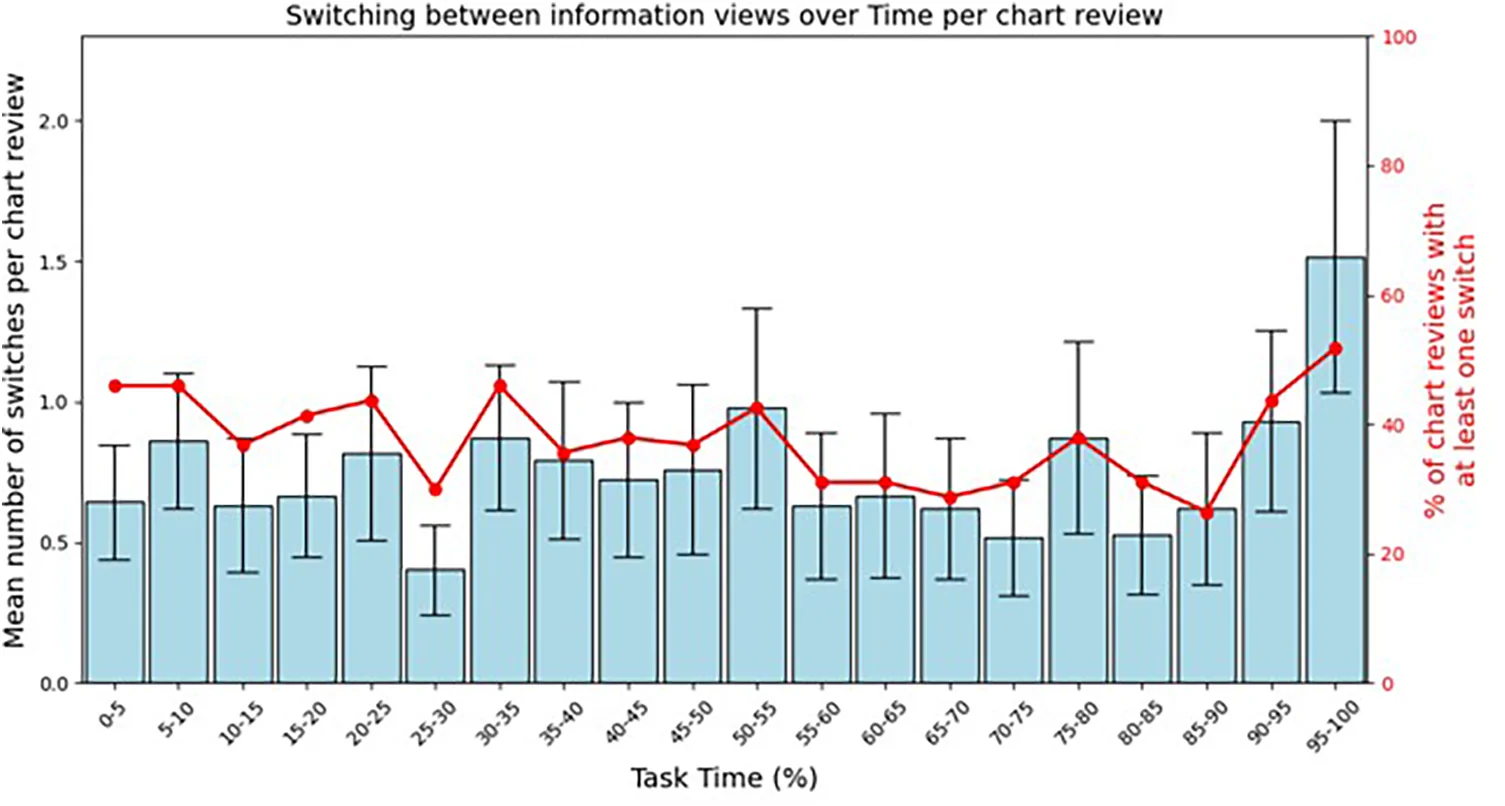
Mean number of switches between information views for every 5% of the chart reviews with 95% confidence intervals (bars, left *Y*-axis), and the percentage of chart reviews with at least one switch (line, right *Y*-axis).

### 3.4 RQ4: opening clinical notes and hyperlinks

The total mean number of opened clinical notes and hyperlinks combined was 11.2 (SD 9.7). Case 1 showed the highest average (16.8, SD 11.1), while Case 2 (9.0, SD 8.1) and Case 3 (7.8, SD 7.3) had lower but comparable levels.

#### 3.4.1 Opened clinical notes

In total, across all three cases, 721 clinical notes were opened for ≥5 s in the clinical notes list during the chart review (Table 4), with a mean of 8.3 (SD 7.3) notes per chart review. The most opened types of notes were from the GP, discharge/outpatient summaries, and nurse notes, with a mean of 1.7, 1.3, and 1.3, respectively. There were some variations between the cases that reflect different clinical contexts.

**Table 4:** Mean number of clinical note types and total clinical notes opened for ≥5 s, all cases combined, and per case. Sorted by the type of note used most frequently across all cases.

Category	All cases (*N* = 87)	Case 1 (*N* = 29)	Case 2 (*N* = 29)	Case 3 (*N* = 29)
General practitioner notes	1.7 (2.2), 0–8	2.4 (2.5), 0–8	1.9 (2.1), 0–7	0.9 (1.5), 0–5
Nursing notes	1.3 (1.9), 0–8	0.0 (0.2), 0–1	1.7 (1.8), 0–6	2.1 (2.2), 0–8
Discharge/outpatient summaries	1.3 (2.1), 0–9	3.3 (2.5), 0–9	0.1 (0.4), 0–2	0.4 (0.8), 0–3
Municipal notes	1.1 (2.1), 0–11	2.8 (2.8), 0–11	0.7 (1.3), 0–5	0.0 (0.0), 0–0
Progress notes	0.8 (1.6), 0–7	0.1 (0.3), 0–1	0.4 (1.0), 0–5	1.8 (2.2), 0–7
Out-of-hours primary care	0.7 (1.4), 0–8	2.0 (1.9), 0–8	0.0 (0.0), 0–0	0.0 (0.0), 0–0
Letters to Patient	0.6 (1.2), 0–6	0.0 (0.2), 0–1	1.9 (1.5), 0–6	0.0 (0.2), 0–1
Lab reports	0.4 (0.9), 0–5	0.2 (0.6), 0–2	0.1 (0.4), 0–2	0.8 (1.4), 0–5
Admission notes	0.3 (0.8), 0–4	0.0 (0.0), 0–0	0.0 (0.2), 0–1	1.0 (1.2), 0–4
Other clinical documents	0.1 (0.3), 0–2	0.1 (0.4), 0–2	0.1 (0.4), 0–2	0.0 (0.0), 0–0
Total (clinical notes)	8.3 (7.3), 0–25	10.9 (7.9), 0–25	7.0 (6.3), 0–22	7.0 (7.3), 0–24

The most used sources were notes from specialist care (4.8 notes), followed by GP (2.4 notes) (Figure 8). Some differences are seen per patient case, in which for Cases 2 and 3, specialist care notes are the most opened (4.4 and 6.1, respectively), whereas the sources were more evenly distributed in Case 1.

**Figure 8:**
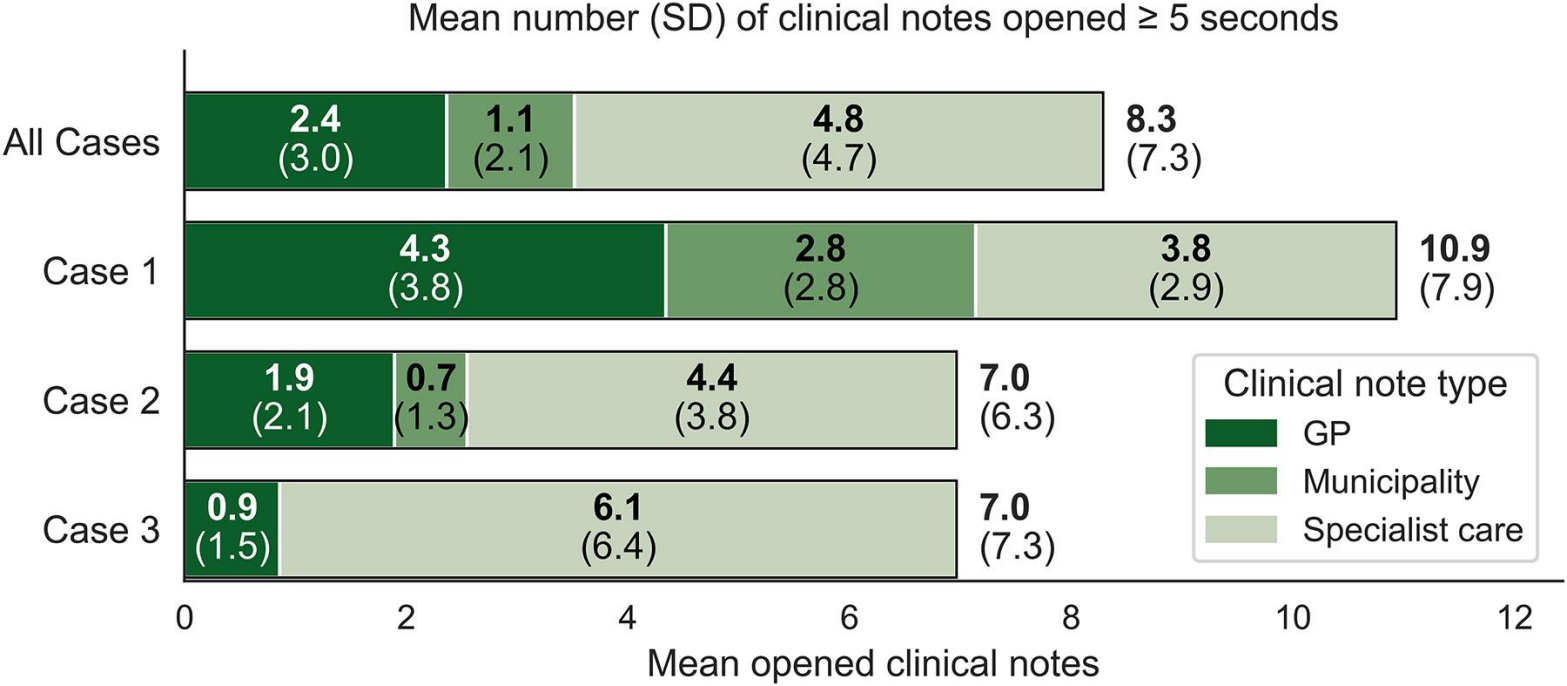
Mean number of opened clinical notes for each of the three sources (GP, municipality, specialist care).

#### 3.4.2 Opened hyperlinks

The “type” of hyperlink refers to the information view in which it was opened (*About the Patient*, *Medical History*, *Medication list*, and *Health Problem Summaries*), not the type of clinical note it linked to.

Across the 87 chart reviews, 252 hyperlinks were clicked on, with an average of 2.9. Hyperlinks in health problem summaries were the most opened, with a mean of 1.7 clicks (Figure 9).

**Figure 9:**
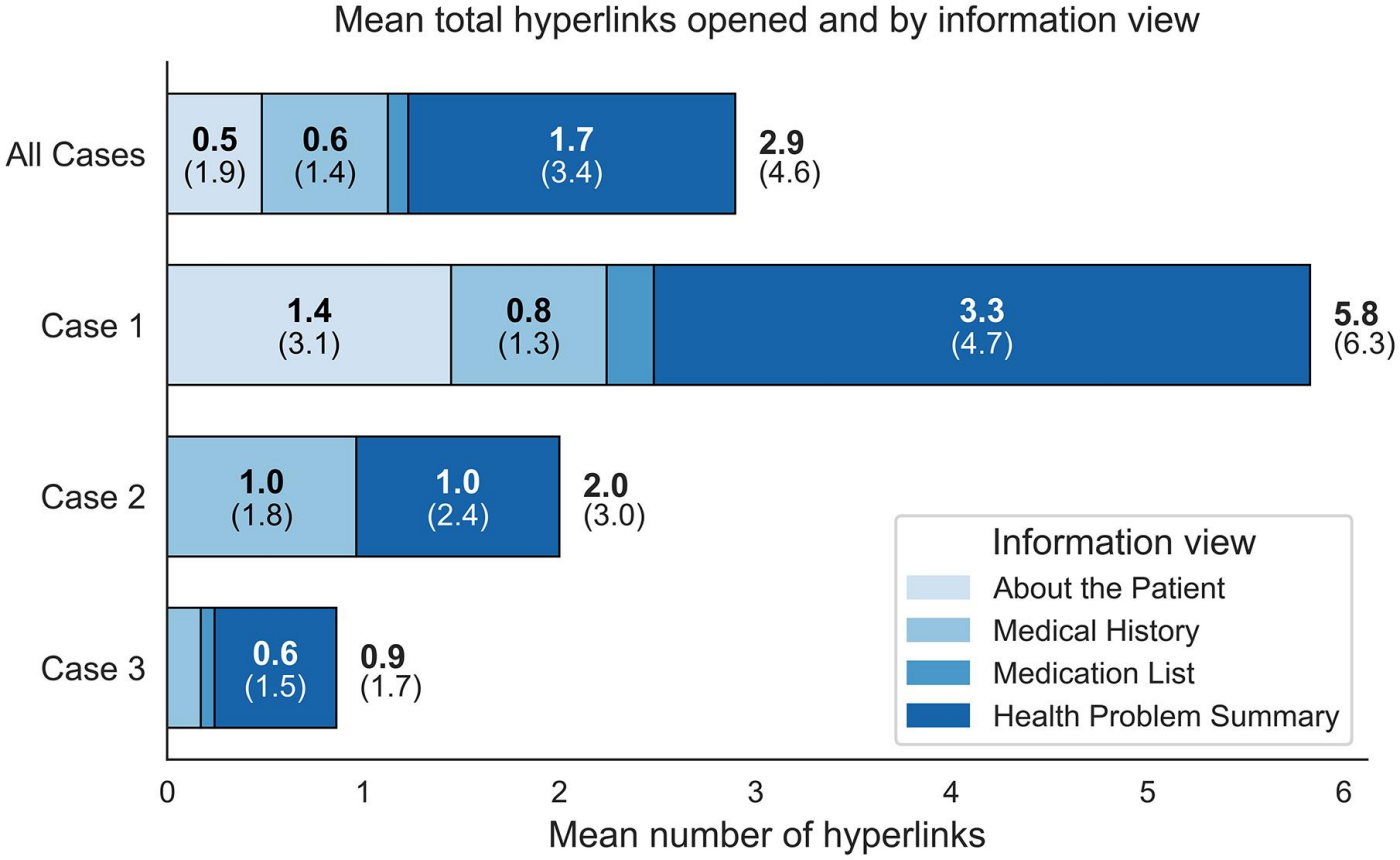
Mean number (SD) of opened hyperlinks for the different information views with hyperlinks for all cases and for each case. Means ≥0.3 are displayed.

The mean number of total opened hyperlinks differed between the cases (respectively 5.8, 2.0, and 0.9) (Figure 9). Hyperlinks in the health problem summaries were most frequently opened in all cases.

## 4 Discussion

### 4.1 Principal findings

This study found that when physicians conduct chart reviews to write a summary of complex patient cases, the participants spent most of their task time in *About the Patient*, followed by *Clinical Notes* and *Health Problem Summaries.* Once DigiTeam was opened and the *Overview page* was displayed, the majority continued to *About the Patient* as their first step in the program, followed by the *Medical History*. Overall, the two most common transitions were from *Overview* to *About the Patient* and from *About the Patient* to *Medical History*.

No studies were found that investigated the usage of a similar digital tool to the one in this study. Therefore, the findings are compared mainly to studies focusing on page usage and navigation patterns when clinicians search for information in EHRs or digital tools for different patient groups and clinical settings. These studies provide context for interpreting the findings on usage and navigation patterns in DigiTeam. However, comparisons between these studies are limited by differences in tasks, interfaces, and clinical context that may affect the patterns of use.

### 4.2 Navigation

The finding that the information views *About the Patient, Medical History,* and *Overview* were opened first, aligns with prior studies showing that a small number of screens are typically used to initiate chart reviews, after which navigation becomes more variable (13). However, previous research has shown that while a limited set of navigation patterns is common, navigation behavior is also influenced by task variation and patient case characteristics (27). Although this study found no substantial differences in page use across patient cases, participants spent more total time on Case 1 than on the other two cases. One possible explanation is that the participants spent more time learning how to use the program during the first case. However, the variation in time spent may also be related to differences in individual case characteristics, or to participant fatigue, as the considerable amount of time spent on Case 1 may have affected the time and effort available for the following cases.

The information on the three most opened information views at the start provided an overview of health problems, key clinical contacts, medical history, what matters to the patient, and social situation. This might reflect the task instructions, which explicitly asked the participants to include specific aspects of the patient's situation. However, both the task and the design of DigiTeam were informed by principles of person-centred, integrated, and proactive care. The observed use may suggest that DigiTeam supported access to an overview of information considered relevant when assessing the patient's situation and planning further care. Furthermore, having this information easily available was said to be useful by the participants during the development and usability testing of DigiTeam (39), where many preferred to begin reading about the patient and reviewing the health problem timeline to identify and prioritize health problems and develop a plan. The *Medical History* was included in DigiTeam at the last stage (39), and was found to be used in this study.

Furthermore, the views chosen first were mainly the same as the ordering of information views in the sidebar menu. This pattern may also be explained by findings from previous studies showing that clinicians often seek summary information to quickly orient themselves when working with large patient records (13, 41) and that clinicians start to search in sections that present the most clinically relevant points, such as the history, problem list, diagnosis, and assessment and plan sections (15, 17, 18, 41, 42). One example of this in this study is that the *Overview* page was primarily used at the beginning, whereas *Health Problem Summaries* were more often used in the middle and later stages. Although the chart reviews started on the Overview page by default, the mean logged duration before navigation to another information view was 2 min 11 s. This pattern suggests that physicians initially orient themselves by reviewing the broader medical history and what matters to the patient, before focusing more specifically on relevant health problems. This aligns with findings during the development of DigiTeam (39) and previous studies (7, 25) reporting that having an “at-a-glance” overview is important for knowing what to search for next, which follows the main design principle used in the development of DigiTeam of “Overview first, zoom and filter, then details on demand” (43).

During the chart review, the physicians moved between information views on average 15 times. Another study found that when providing consolidated information on a single page, the number of switches dropped from 43 to 14 (12). Thus, redesigning EHR displays can reduce cognitive load caused by frequent switches and is also preferred by clinicians compared to interfaces where information is distributed across multiple tabs (44).

### 4.3 Use of information views

The development of DigiTeam was grounded in the importance of presenting information about the patient's social and contextual situation, and what matters most. This information was highly appreciated during the user tests when developing DigiTeam, as it was said to support care planning and prioritization based on patients’ preferences and goals (39). The length of time spent in About the Patient observed in this study aligns with findings from the development of DigiTeam that this information is needed for a comprehensive overview of the patient's situation. At the same time, the log data cannot establish why participants spent longer periods of time in this view, as it could also be influenced by the task and the amount of information presented. Nevertheless, considering the patient's broader context is important when planning care beyond medical needs (45), and this information was for that reason asked to be included during the task in the RCT. The findings showed that when this information was available in a summarized format, participants spent much time on it. Such a detailed summary of contextual information about the patient has not been identified in previous studies. Although not directly comparable, other studies have found that clinicians engage less frequently with sections for social information compared to other types of information in the EHR (15, 46). Studies report barriers in EHRs to document and retrieve such information about the patient (47–49), and that social and contextual information are not always well integrated into clinical workflows (46). This indicates a need for better presentation of such patient information in EHRs.

The *Clinical Notes List* was the second most used page, accounting for 20% of total time. Other studies have found that, of the total EHR time, clinicians spend from 17% in primary care settings (2) up to nearly half in intensive care and specialist care contexts (19, 24). In this study, physicians viewed an average of eight notes, and the most frequently opened were GP notes and discharge and outpatient summaries, which are known to provide high-yield clinical information (41).

Despite this, both the proportion of time spent and the number of notes opened in DigiTeam were relatively low, given the large volume of available documentation in the cases. This may indicate that physicians found relevant information elsewhere, as reflected in the high use of other information views with integrated summaries. This aligns with a study finding that physicians used summaries alone to answer 70% of clinical questions during a test, although having access to clinical notes (35). Several studies report on summary-based solutions to support chart review, and similar to what was found in the development of DigiTeam (39), report positive user feedback (7, 29, 35, 50). Furthermore, consistent with earlier studies, hyperlinks can enhance trust in summarized information by allowing users to verify information with the original documents (7, 35, 39). This suggests that, aligned with previous studies (7, 35), summaries with the possibility to check the original notes are a valuable information element.

### 4.4 Strengths and limitations

The main strength of this study is that it is, to our knowledge, the first to investigate the actual usage of a digital tool like DigiTeam using log data. Log data were collected during an RCT, where all physicians received the same instructions and settings. Although the instructions were intended to reflect actual clinical practice, the task instructions may have impacted the use of DigiTeam. Furthermore, the study was conducted in an experimental setting, which differs from real clinical practice, and this difference can impact the way information is sought. DigiTeam also did not include interactive functionalities that are found in EHR systems used in clinical practice and part of clinical workflow (e.g., order entry and management, documentation, medication prescription, secure messaging, etc). This simplification can influence total time spent but probably has little influence on patterns of use. DigiTeam was introduced with only a short instruction video, and with more time and experience, clinicians may have used it differently. The type of participants included, general practitioners and physicians with little experience, means that the findings might not be generalizable to other clinical settings.

Using log data only showed when an information view was opened and switched to another one, and cannot distinguish between intentional or accidental switches, but this is considered a limited problem. Furthermore, such log data do not reveal what information clinicians actually read, which elements they focused on, why pages were opened, or how much time was spent writing the summary note.

## 5 Conclusion

When reviewing complex patient cases, physicians mainly used information views that summarize and visualize patient information, and read clinical notes. Most used information views included information on the patient's context, social situation, what matters to the patient, the medical history, and specific health problems. These findings, based on actual use rather than perceived usefulness, suggest that integrating such elements within digital tools may better support chart review for patients with complex and long-term care needs.

## Data Availability

The raw data supporting the conclusions of this article will be made available by the authors, without undue reservation.
